# Blood Hemoglobin Substantially Modulates the Impact of Gender, Morbid Obesity, and Hyperglycemia on COVID-19 Death Risk: A Multicenter Study in Italy and Spain

**DOI:** 10.3389/fendo.2021.741248

**Published:** 2021-11-02

**Authors:** Jordi Mayneris-Perxachs, Maria Francesca Russo, Rafel Ramos, Ana de Hollanda, Arola Armengou Arxé, Matteo Rottoli, María Arnoriaga-Rodríguez, Marc Comas-Cufí, Michele Bartoletti, Ornella Verrastro, Carlota Gudiol, Ester Fages, Marga Giménez, Ariadna de Genover Gil, Paolo Bernante, Francisco Tinahones, Jordi Carratalà, Uberto Pagotto, Ildefonso Hernández-Aguado, Fernando Fernández-Aranda, Fernanda Meira, Antoni Castro Guardiola, Geltrude Mingrone, José Manuel Fernández-Real, Obesity-T2DM Covid19 Study Group, Mariona Reixach Fuma&ntilde;a

**Affiliations:** ^1^ Department of Diabetes, Endocrinology and Nutrition, Dr. Josep Trueta University Hospital, Girona, Spain; ^2^ Nutrition, Eumetabolism and Health Group, Girona Biomedical Research Institute (IdibGi), Girona, Spain; ^3^ Department of Endocrinology and Nutrition, Centro de Investigación Biomédica en Red-Fisiopatología de la Obesidad y Nutrición (CIBEROBN), Madrid, Spain; ^4^ Fondazione Policlinico Universitario A. Gemelli Istituto di Ricovero e Cura a Carattere Scientifico (IRCCS), Università Cattolica del Sacro Cuore Rome, Rome, Italy; ^5^ Department of Medical Sciences, School of Medicine, University of Girona, Girona, Spain; ^6^ Vascular Health Research Group of Girona (ISV-Girona). Jordi Gol Institute for Primary Care Research (Institut Universitari per a la Recerca en AtencióPrimària Jordi Gol I Gurina -IDIAPJGol), Girona Biomedical Research Institute (IDIBGI), Dr. Josep Trueta University Hospital, Catalonia, Spain; ^7^ Girona Biomedical Research Institute (IDIBGI), Dr. Josep Trueta University Hospital, Catalonia, Spain; ^8^ Department of Endocrinology & Nutrition, Hospital Clínic Barcelona, Diabetes Unit, Barcelona, Spain; ^9^ Department of Endocrinology and Nutrition, Institut d’investigacions biomèdiques August Pi i Sunyer (IDIBAPS), Barcelona, Spain; ^10^ Department of Internal Medicine, Service of Internal Medicine Dr. Josep Trueta University Hospital, Catalonia, Spain; ^11^ Department of Surgery, Diabetology and Otolaryngology, Surgery of the Alimentary Tract and Centre for the Study and Research of Treatment for Morbid Obesity, Bologna, Italy; ^12^ Infectious Diseases Unit, Department of Medical and Surgical Sciences, Alma Mater Studiorum University of Bologna, Sant’Orsola Policlinic, Bologna, Italy; ^13^ Department of Infectious Diseases, Hospital Universitari de Bellvitge, Hospitalet de Llobregat, Barcelona, Spain; ^14^ Bellvitge Biomedical Research Institute (IDIBELL), Hospitalet de Llobregat, Barcelona, Spain; ^15^ Spanish Network for Research in Infectious Disease (REIPI), Instituto de Salud Carlos III, Madrid, Spain; ^16^ Department of Clinical Sciences, School of Medicine and Health Sciences, University of Barcelona, Barcelona, Spain; ^17^ Department of Oncology, Institut Català d’Oncologia (ICO) Hospitalet de Llobregat, Barcelona, Spain; ^18^ Department of Endocrinology and Nutrition, Centro de Investigación Biomédica en Red de Diabetes y Enfermedades Metabólicas Asociadas (CIBERDEM), Madrid, Spain; ^19^ Department of Endocrinology and Nutrition, Hospital Clínico Universitario Virgen de Victoria de Malaga, Malaga, Spain; ^20^ Endocrinology and Prevention and Care of Diabetes Unit, Department of Medical and Surgical Sciences, Sant’ Orsola Policlinic, Alma Mater Studiorum University of Bologna, Bologna, Italy; ^21^ Department of Public Health, Universidad Miguel Hernández de Elche, Alicante, Spain; ^22^ Department of Epidemiology, Centro de Investigación Biomédica en Red (CIBER) Epidemiology and Public Health (CIBERESP), Madrid, Spain; ^23^ Department of Psychiatry, Hospital Universitari de Bellvitge, Hospitalet de Llobregat, Barcelona, Spain; ^24^ Department of Infectious Diseases, Hospital Clínic Barcelona, Barcelona, Spain; ^25^ Department of Internal Medicine, King’s College London, London, United Kingdom

**Keywords:** COVID-19, hemoglobin, hyperglycemia, obesity, epidemiology, mortality, machine learning

## Abstract

**Background:**

Hyperglycemia and obesity are associated with a worse prognosis in subjects with COVID-19 independently. Their interaction as well as the potential modulating effects of additional confounding factors is poorly known. Therefore, we aimed to identify and evaluate confounding factors affecting the prognostic value of obesity and hyperglycemia in relation to mortality and admission to the intensive care unit (ICU) due to COVID-19.

**Methods:**

Consecutive patients admitted in two Hospitals from Italy (Bologna and Rome) and three from Spain (Barcelona and Girona) as well as subjects from Primary Health Care centers. Mortality from COVID-19 and risk for ICU admission were evaluated using logistic regression analyses and machine learning (ML) algorithms.

**Results:**

As expected, among 3,065 consecutive patients, both obesity and hyperglycemia were independent predictors of ICU admission. A ML variable selection strategy confirmed these results and identified hyperglycemia, blood hemoglobin and serum bilirubin associated with increased mortality risk. In subjects with blood hemoglobin levels above the median, hyperglycemic and morbidly obese subjects had increased mortality risk than normoglycemic individuals or non-obese subjects. However, no differences were observed among individuals with hemoglobin levels below the median. This was particularly evident in men: those with severe hyperglycemia and hemoglobin concentrations above the median had 30 times increased mortality risk compared with men without hyperglycemia. Importantly, the protective effect of female sex was lost in subjects with increased hemoglobin levels.

**Conclusions:**

Blood hemoglobin substantially modulates the influence of hyperglycemia on increased mortality risk in patients with COVID-19. Monitoring hemoglobin concentrations seem of utmost importance in the clinical settings to help clinicians in the identification of patients at increased death risk.

## 1 Introduction

Since the first reports from China at the beginning of the COVID-19 pandemic, age, male sex, obesity, type 2 diabetes mellitus (T2D), arterial hypertension (AHT) and cardiovascular disease have been identified as established risk factors for a poor prognosis in patients with SARS-CoV-2 infection (1). Initial multivariate analysis confirmed that individuals with BMI > 40 kg/m^2^ had 2.5 (1.8-3.4) (odds ratio and 95% confidence interval) times more risk for hospital admission, and those with grade II obesity had 7.36 (1.6-33) times more requirement of invasive mechanical ventilation, compared with normal-weight COVID-19 patients (1–3).

Different systematic reviews have substantiated that subjects with obesity are at higher risk for hospitalization, ICU admission and mortality (4, 5). However, these studies did not mention the detailed comorbidities of patients with obesity, which may confound the role of obesity as an independent risk factor in COVID-19 (6). For instance, in a community-based cohort study, both obesity and central obesity had an upward linear trend with the COVID-19 hospitalization that was attenuated after adjustment for confounding factors. For example, HbA1c and HDL cholesterol attenuated the association by 33% and 46%, respectively (7). In addition, the different results across the studies can be simply justified by the fact that many of them did not analyze laboratory parameters, but just the demographic characteristics of patients.

On the other hand, the prevalence of diabetes among patients with COVID-19 varied from 6% to more than 22% in subjects with mild disease or severe forms, respectively, and raised to more than 30% among subjects requiring ICU admission (1, 8). In a retrospective, multi-centered study of 7,337 cases in HubeiT2D patients required more medical interventions, had a significantly higher mortality (7.8% versus 2.7%; adjusted hazard ratio (HR), 1.49), and multiple organ injury than non-diabetic individuals. Patients with well-controlled blood glucose concentration (glycemic variability within 3.9 to 10 mmol/L) had markedly lower mortality compared to individuals with poorly controlled blood glucose (upper limit of glycemic variability exceeding 10.0 mmol/L) (adjusted HR=0.14) during hospitalization. However, the results were not adjusted by the potential confounding effects of adiposity (9). Other studies have found diverging effects of impaired fasting glucose and T2D diagnosis on COVID-19 prognosis (10). Hyperglycemia in the acute phase also predicted worse outcomes in hospitalized patients with COVID-19. For instance, a study of 184 hospitalized patients showed that severe COVID-19 occurred in the presence of both impaired glucose metabolism and obesity (11). However, most of the studies reporting an association between hyperglycemia and a poorer prognosis did not take into account the confounding effects of obesity status, whereas studies assessing obesity status on COVID-19 did not include comorbidities such as diabetes (12–16).

Besides considering the joint role of obesity and diabetes in modifying COVID-19 outcomes, it is vital to identify those factors that modulate the impact of both obesity and hyperglycemia on COVID-19 prognosis. For example, the levels of albumin, hemoglobin, the mean platelet volume, and inflammatory markers (monocyte to eosinophil or neutrophil to lymphocyte ratios) are prognostic markers in patients with COVID-19 (17–19). In particular, anemia has been shown to be an independent predictor of mortality in COVID-19 patients (20–22). Therefore, we hypothesized that the inclusion of inflammatory and hematological markers would modulate the associations of obesity and hyperglycemia with death and ICU admission. We first aimed to evaluate the joint role of obesity and diabetes in modifying COVID-19 outcomes. Then, we applied a machine learning algorithm to identify additional confounding factors with potential impact on modulating the obesity and hyperglycemia prognostic values.

## 2 Materials and Methods

### 2.1 Outcomes

The primary outcomes were all-cause mortality due to COVID-19 and admission to an ICU. For ICU and mortality risk, only hospital-admitted patients were evaluated. Consecutive unselected patients who were admitted to the hospital and diagnosed with pneumonia by SARS-CoV-2 were included. In Primary Health care centers, all consecutive patients fulfilling inclusion criteria were also analyzed. Secondary outcomes included clinical routine variables such as age, sex, glucose, BMI, SBP and creatinine. Finally, tertiary outcomes included hemoglobin, bilirubin, platelet counts, sodium levels, and hs-CRP.

### 2.2 Inclusion Criteria of the Different Hospitals and Centers

**Figure 1:**
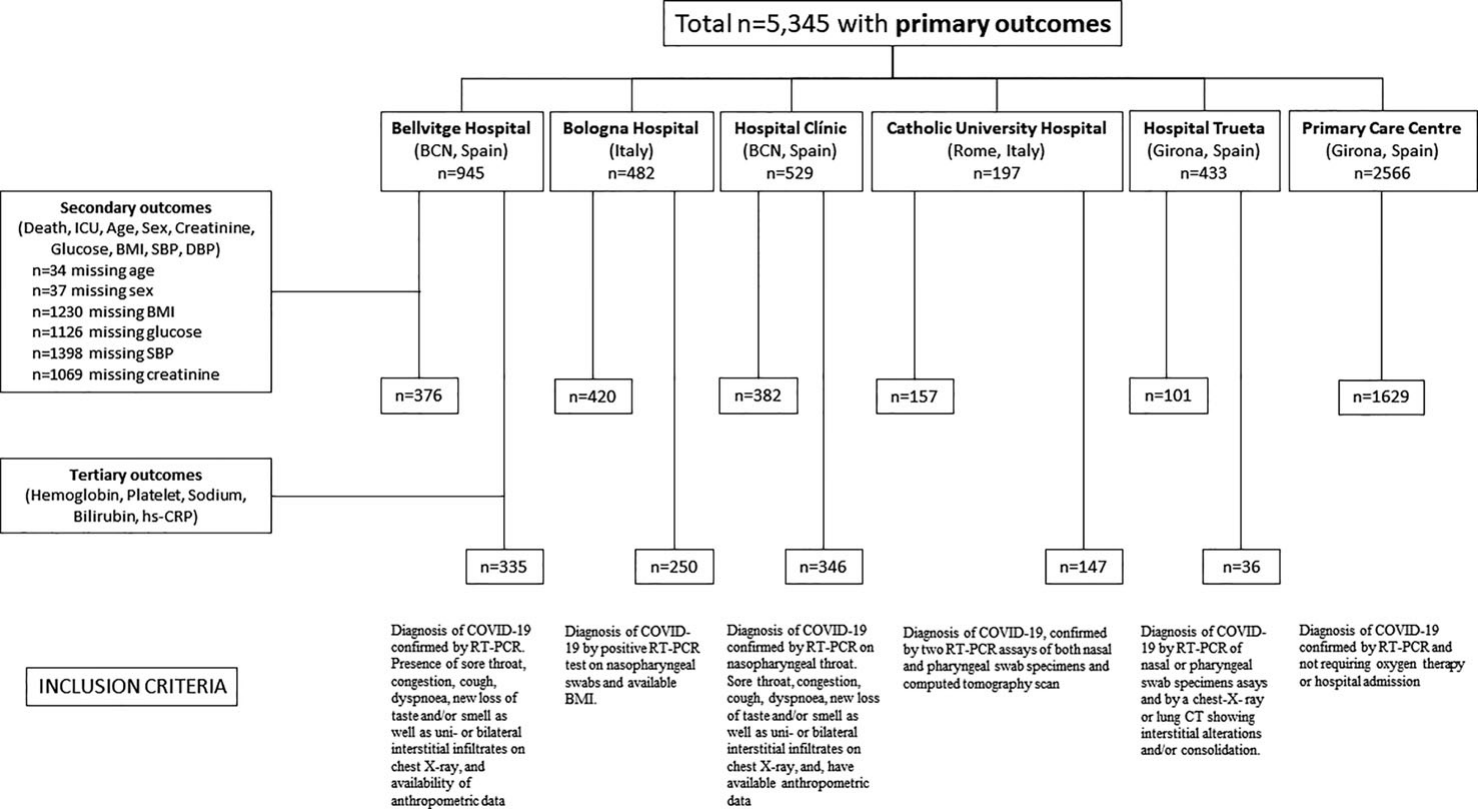
Flow chart of study design and inclusion criteria.

A description of the study design and the inclusion criteria can be found in **Figure 1**. We had available data from patients of five different hospitals and participants from one primary care center with a diagnosis of COVID-19 (*n=*5,345). From these patient, *n*=3,065 had available data on both the primary and secondary outcomes. In addition, a subset of these patients (*n*=1,114) had also available data about the tertiary outcomes, including hemoglobin (*n*=1445), platelet counts, sodium levels, bilirubin or hs-CRP.

#### 2.2.1 Catholic University Hospital of Rome

All patients admitted to the hospital between March 1st and May 1st, 2020 were included. Inclusion criteria for admission at the Fondazione Policlinico Gemelli IRCCS in Rome, Italy, were diagnosis of pneumonia from SARS-CoV-2, confirmed by two consecutive real-time reverse-transcriptase polymerase-chain-reaction (RT-PCR) assays of both nasal and pharyngeal swab specimens and computed tomography (CT) scan.

#### 2.2.2 Hospital of Bologna

Consecutive patients admitted to the hospital between March 1 and April 20, 2020were included. The last follow-up date was April 27, 2020. Inclusion criteria: patients who had a confirmed COVID-19 diagnosis using a positive RT-PCR (Reverse transcription polymerase chain reaction) test on nasopharyngeal swabs and available BMI.

#### 2.2.3 Bellvitge University Hospital

Patients consecutively admitted to Bellvitge University Hospital with laboratory-confirmed COVID-19 infection by RT-PCR assay for SARS-CoV-2 between March and May, 2020 were included. The inclusion criteria were the presence of sore throat, congestion, cough, dyspnoea, new loss of taste and/or smell, as well as uni- or bilateral interstitial infiltrates on chest X-ray, and availability of anthropometric data.

#### 2.2.4 Hospital Clínic, Barcelona

All patients admitted with COVID-19 for ≥48 hours between 28 February and 22 April 2020 were included. All patients had a diagnosis of COVID-19 confirmed by real-time reverse transcription PCR (RT-PCR) testing performed on nasopharyngeal throat swab specimens, and/or by fulfilling clinical diagnostic criteria provided during the pandemic peak for SARS-CoV-2. These criteria comprised the presence of any of the following respiratory symptoms: sore throat, congestion, cough, dyspnoea, new loss of taste and/or smell as well as uni- or bilateral interstitial infiltrates on chest X-ray, and, have available anthropometric data registered up to one year before admission.

#### 2.2.5 Hospital of Girona

Consecutive adult patients hospitalized between March 14, and June 30, 2020 with confirmed COVID-19 pneumonia defined as a positive result on real-time reverse-transcriptase polymerase-chain-reaction (RT-PCR) of nasal or pharyngeal swab specimens assays and also by a chest-X- ray or lung CT showing interstitial alterations and/or consolidation were included. The criterion for patients’ admission to ICU was the failure to conventional oxygen therapy delivered through a non-rebreathing mask requiring a most intensive form of ventilatory support.

#### 2.2.6 Girona Primary Care participants

All the individuals with confirmed COVID-19 (defined as having a positive RT-PCR test for SARS-CoV-2 virus) and attended in the general practices in Girona patients in whom management did not require oxygen therapy or hospital admission.

### 2.3 Study Variables

The data gathered for all cohorts were obtained from the electronic clinical health records, registered previously to the COVID19 diagnosis (in primary health care centers) or during hospital admission. Patient confidentiality was protected by assigning an anonymous identification code, anonymously discharged, and included the following: age, sex, systolic and diastolic blood pressure, body mass index, vascular risk factors (diabetes mellitus, hypertension, obesity, hypercholesterolemia, smoking) and laboratory tests (blood hemoglobin, serum bilirubin, total cholesterol, LDL-cholesterol, HDL-cholesterol, triglycerides, creatinine, and fasting glucose, among others). In all cases, glycemia and other analytes were measured using routine laboratory analyses in fasting conditions within 24 hours of hospital admission. All patients were followed until discharge or until death.

### 2.4 Ethics Committee’ Approvals

The protocol was approved by the Ethical Committee of the Fondazione Policlinico Universitario A. Gemelli IRCCS, Catholic University, Rome, Italy with Approval Number: 0014355/20. Before enrolment, each subject gave informed consent. ClinicalTrials.gov ID: NCT04324684. The protocol was also independently approved by the ethics committee of the Hospital of Bologna, Hospital of Bellvitge, Hospital Clínic, Hospital of Girona and the Primary Care Ethics Committee.

### 2.5 Statistical Analysis

We examined the associations among potential prognostic variables and the main outcomes using multivariate logistic regression models. We used the glm function in R with a binomial family, a logit link function, and the Wald test to estimate the p-values of the model parameters. Odds ratios were then obtained as the exponentials of the parameters estimates and 95% confidence intervals (CI) were computed using the “confint” function from the MASS package. Age was categorized in three groups [<50 (50,70), and >70 years], SBP was dichotomized [(0,130), ≥130 mmHg], obesity was categorized according to the BMI [underweight, <18.5; normal range, (18.5,25); overweight, (25,30); obese class I, (30,35), obese class II, (35,40), obese class III, ≥40 kg/m2] and glycemia status at hospital admission (generally in the first 24 hours after overnight fasting) or in fasting conditions in primary health care centers. It was categorized based on fasting blood glucose levels [hypoglycemia, <70; normal, (70,100); moderate hyperglycemia, (100,126); severe hyperglycemia, (126,200); extreme hyperglycemia, ≥200]. Creatinine was categorized as below or above 1.3 mg/dl. For the subset of patients with complete data on all clinical variables (*n*=1,114), we further analyzed the data dichotomizing iron-related parameters (hemoglobin and bilirubin) based on the median for each gender and building logistic regression models for each group and gender. The performance of these models in classifying patients (predictive ability) was evaluated using the area under the receiver operating characteristics (AUCROC) curve. In addition, we further validated the classification prediction accuracy of these models by external validation. A total of *n*=1,445 patients had available data on age, sex, SBP, BMI, glucose and hemoglobin levels. Therefore, we used the *n*=331 samples not used in training the models as a test set and predicted the main outcome (death or survival) in this test set using the models for both low and high hemoglobin levels. We used a probability threshold of 0.5 to classify samples in one group (death) or another (survival) and we assessed the model accuracy as the proportion of samples that had been correctly classified.

### 2.6 Machine Learning

In addition to using multivariate logistic regression models, we also applied a machine learning algorithm (Boruta) to the subset of samples with complete (primary, secondary and tertiary outcomes) clinical data (*n*=1,114) to identify the most relevant clinical variables related to both death and ICU admission. The Boruta algorithm is a wrapper algorithm that performs feature selection based on the learning performance of the model. It has been recently proposed as one of the two best-performing variable selection methods making use of random forests (23). It performs variables selection in four steps: a) Randomization. To create a duplicate copy of the original features randomly permutate across the observations (the so-called shadow features) to remove their correlation with the response; b) Model building. To add the shadow feature to the original predictor feature data set, built a RF with the extended data set, and compute the normalized permutation importance (Z) scores for each predictor and shadow feature; c) Statistical testing. To find the maximum normalized importance among the shadow attributes (MZSA) and compare it with each original predictor feature using a Bonferroni corrected two-tailed binomial test. Predictor features with significantly higher, significantly lower, or non-significantly different Z scores than expected at random compared to the MZSA are deemed important, unimportant, or tentative, respectively. d) Iteration. Unimportant and shadow features are removed and the previous steps are repeated until the status of all features is decided or a predefined number of iterations has been performed.

We run the Boruta algorithm with 1000 iterations, a confidence level cut-off of 0.005 for the Bonferroni adjusted p-values, 2000 trees to grow the forest (ntree), and several features randomly sampled at each split given by the square root of the number of features (the mtry recommended for discriminant analysis).

## 3 Results

**Figure 2:**
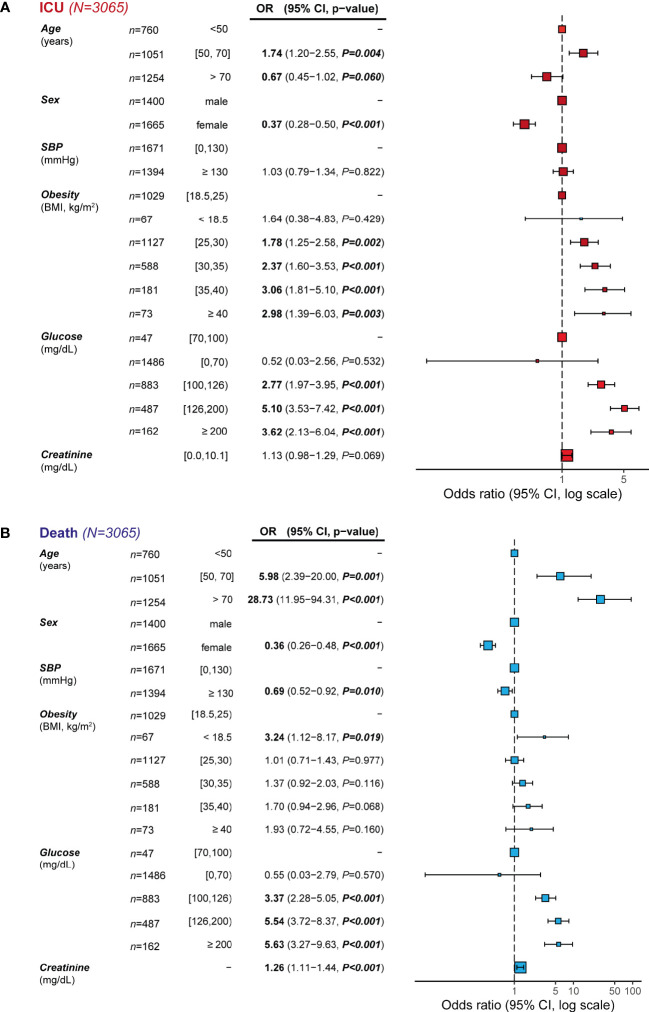
Incidence odds ratio of **(A)** admission to the ICU and **(B)** mortality from COVID-19 by potential prognostic variables. Data are presented as odds ratio (OR) and 95% confidence intervals (CI). The reference groups (OR=1) for each variable are shown as “-”.: The odds ratios shown are adjusted through a logistic regression model which includes all variables listed. Data are also represented as a forest plot.

Out of 5,345 patients included in the database with available data on the primary outcomes, 3,065 had all data available for the secondary outcomes for further analyses (**Table 1**). Within subjects admitted to the hospital, both obesity and hyperglycemia were independent predictors of admission in the ICU (**Figure 2A**). Hence, while subjects with obesity were 2-3 times more likely to be admitted to the ICU compared to normal weight individuals, subjects with hyperglycemia were 3.5-5 times more likely to enter the ICU than normoglycemic individuals. In line with the death incidence, men were about 63% more likely to be admitted to the ICU than women.

**Table 1:** Characteristics of the patients included in each Institution.

	Bellvitge (N=376)	Bologna (N=420)	Clinic (N=382)	Trueta (N=101)	NIKE (N=157)	Primary Care (N=1629)	Total (N=3065)
**Death**	88 (23.4%)	83 (19.8%)	60 (15.7%)	9 (8.9%)	26 (16.6%)	0 (0.0%)	266 (8.7%)
**ICU**	48 (12.8%)	51 (12.1%)	154 (40.3%)	20 (19.8%)	17 (10.8%)	0 (0.0%)	290 (9.5%)
**Age** (years)	68.0 (56.0,76.0)	68.0 (55.0,80.0)	64.0 (54.0,73.0)	62.0 (48.0,73.0)	65.0 (54.0,76.0)	61.9 (43.8,84.6)	65.0 (49.4,80.0)
**Sex**	151 (40.2%)	160 (38.1%)	148 (38.7%)	44 (43.6%)	59 (37.6%)	1103 (67.7%)	1665 (54.3%)
**Creatinine** (mg/dL)	0.9 (0.7, 1.2)	0.9 (0.8, 1.1)	0.9 (0.8, 1.1)	0.7 (0.6, 1.0)	0.9 (0.8, 1.2)	0.8 (0.7, 1.0)	0.8 (0.7, 1.0)
**Glucose** (mg/dL)	113.5 (100.9,140.6)	111.0 (99.0,132.2)	110.0 (97.0,131.8)	117.0 (105.0,140.0)	110.0 (98.0,128.0)	90.0 (81.0,101.0)	99.1 (86.5,119.0)
**BMI** (kg/m^2^)	28.9 (26.0, 31.6)	25.1 (23.0,28.4)	27.8 (24.9,31.2)	31.2 (27.0, 38.0)	25.4 (24.1,27.3)	26.2 (23.0,29.9)	26.7 (23.7,30.3)
**SBP** (mmHg)	130.5 (117.0, 43.0)	125.0 (111.5,135.0)	124.0 (112.0,140.0)	131.0 (119.0, 144.0)	125.0 (115.0,140.0)	127.0 (116.0,136.0)	127.0 (115.0, 38.0)
**DBP** (mmHg)	72.0 (64.0, 81.0)	70.0 (70.0, 80.0)	72.0 (65.0, 81.0)	74.0 (65.0, 85.0)	79.0 (70.0, 85.0)	74.0 (67.0, 80.0)	73.0 (66.0, 80.0)

Results from multivariate logistic regression analysis among prognostic variables and the incidence of mortality from COVID-19 are shown in **Figure 2B**. We identified age as the main independent predictor of mortality, with subjects over 70 years having a 29 times higher likelihood of presenting death due to COVID-19 than subjects younger than 50. Sex, SBP, glucose and creatinine were also independent predictors of death. Hence, men were about 64% more likely to present death due to COVID-19 than women. A unit increase in the standard deviation of plasma creatinine levels was associated with a 26% increase in mortality due to COVID-19. Remarkably, while subjects with glycemia>100 (moderate hyperglycemia) and >126 mg/dl (severe hyperglycemia) had 3.5 and 5.5 times higher mortality risk than normoglycemic subjects, no differences were found in COVID-19 mortality by weight categories. Only underweight, BMI < 18.5 kg/m^2^, was associated with a higher mortality risk. We also considered the potential interaction between some of the predictor variables in the models. However, we did not find any significant interaction between age and sex (P=0.274), age and SBP (P=0.710), age and BMI (P=0.131), age and glucose (P=0.774), glucose and SBP (P=0.567), glucose and BMI (P=0.599), or BMI and SBP (P=0.309).

These results were replicated considering only patients admitted to the hospital in each country independently (**Supplementary Figures 1A, B** for mortality and ICU in Spain and **Supplementary Figures 3C, D** for mortality and ICU in Italy, respectively). The addition of previously known T2D to the models did not significantly change any of the previous results.

Finally, when the results were compared between patients recruited in primary care and those admitted to hospitals, there were no appreciable differences in the magnitude of the associations.

### 3.1 Machine Learning Results

**Figure 3:**
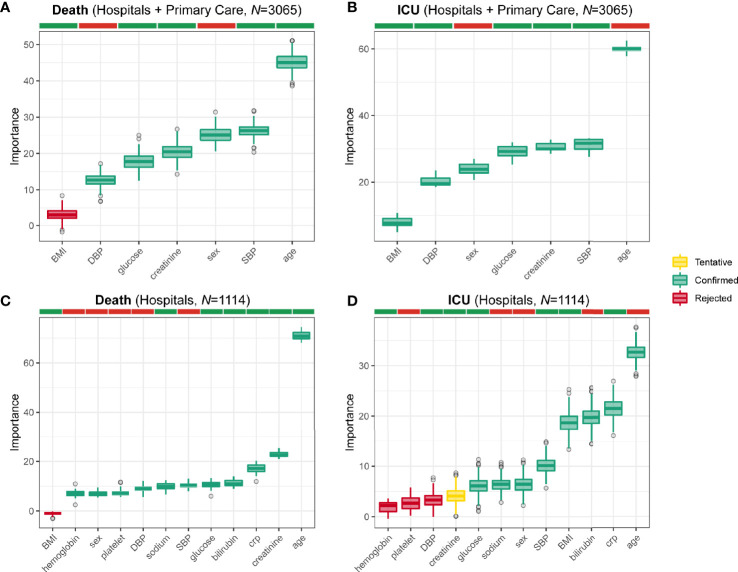
Mortality and ICU admission prognostic variables identified from machine learning. Boxplots of the normalized permutation importance obtained from the Boruta algorithm for the potential prognostic variables associated to the **(A)** mortality from COVID-19 in all subjects (*n*=3065), **(B)** admission to the ICU in all subjects (*n*=3065), **(C)** mortality from COVID-19 in hospital patients (*n*=1114), and **(D)** admission to the ICU in hospital patients (*n*=1114).

We further analyzed the data applying a machine learning variable selection strategy based on multiple random forest as implemented in the Boruta algorithm (24) to identify the most important outcome predictive variables. In line with the previous analysis, age was the strongest predictor of both mortality (**Figure 3A**) and ICU admission (**Figure 3B**). Notably, BMI discriminated those subjects admitted to the ICU from subjects who did not need admission, but was unable to discriminate between individuals who died due to COVID-19 from those who survived, thereby corroborating the results from the logistic regression models. In the case of hospital patients, we were able to include additional variables to the machine learning algorithms. From the total of 1,446 admitted to the hospital, 1,114 also had measurements of plasma C-Reactive Protein (CRP), bilirubin, sodium, platelet, and hemoglobin levels. Once again, age was the most differentiating variable in the case of death (**Figure 3C**) and ICU (**Figure 3D**) admission outcomes, whereas BMI was only able to discriminate among ICU patients (**Figure 3D**). It is worth noting that bilirubin, CRP, and creatinine had a strong association with death, but only bilirubin and CRP were also associated with ICU admission. Hyperglycemia was independently associated with a poorer prognosis.

**Figure 4:**
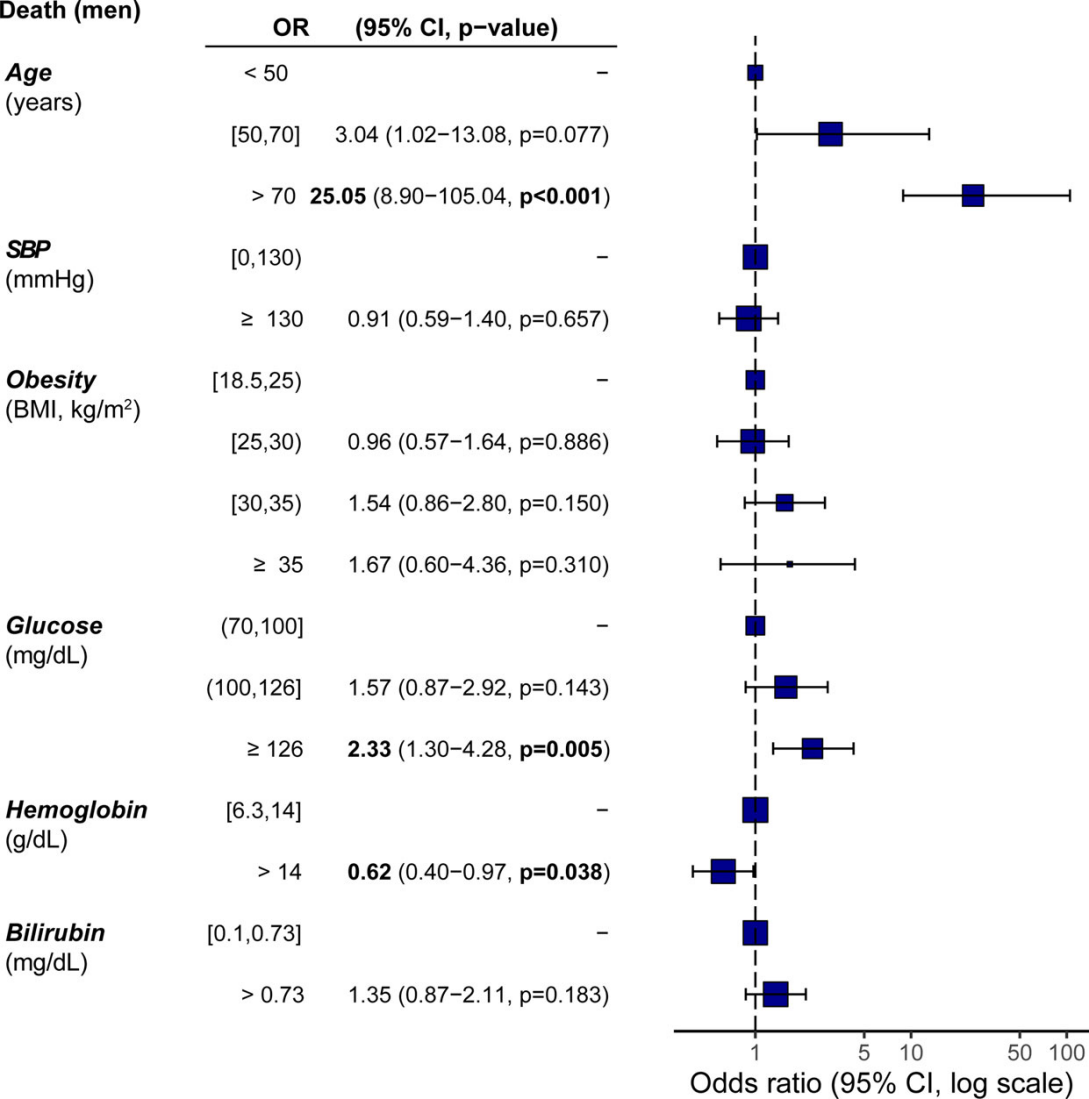
Incidence odds ratio of mortality from COVID-19 in men including iron-related parameters as prognostic variables. Data are presented as odds ratio (OR) and 95% confidence intervals (CI). The reference groups (OR=1) for each variable are shown as “-”.: The odds ratios shown are adjusted through a logistic regression model which includes all variables listed. Data are also represented as a forest plot. Hemoglobin and bilirubin were dichotomized based on the median values.

The machine learning strategy identified two iron-related parameters (hemoglobin and bilirubin) as significant predictors of death. Therefore, we built new multivariate logistic regression models including hemoglobin and bilirubin dichotomized based on their median values. As both parameters are strongly dependent on gender, separate analyses were also performed for men and women. We found that hemoglobin and bilirubin were not predictors of mortality in women. However, hemoglobin had a significant prognosis effect in the case of men (**Figure 4**). Men having hemoglobin values above the median were less likely to die due to COVID-19 than men with concentrations below the median. Remarkably, glucose was a strong predictor of mortality in men with hemoglobin or bilirubin values above the median, but not in those with concentrations below the median (**Supplementary Figure 2**). Therefore, men with moderate or severe hyperglycemia that also had blood hemoglobin concentrations over the median had about 17 and 30 times increased mortality risk compared to men without hyperglycemia, respectively (**Supplementary Figure 2B**), whereas there were no significant differences in mortality among men with low hemoglobin values (**Supplementary Figure 2A**). Similarly, morbidly obese men also had a higher mortality risk than normo-weight individuals only in those individuals with hemoglobin levels above the median (**Supplementary Figure 2**). Likewise, glucose concentration was not a prognosis predictor of mortality in men with bilirubin values below the median (**Supplementary Figure 2C**), but had a strong impact in men with bilirubin concentrations above the median (**Supplementary Figure 2D**). Women with hyperglycemia and increased blood hemoglobin also had a trend towards mortality risk compared with women without hyperglycemia (OR=3.17, *P*=0.096).

**Figure 5:**
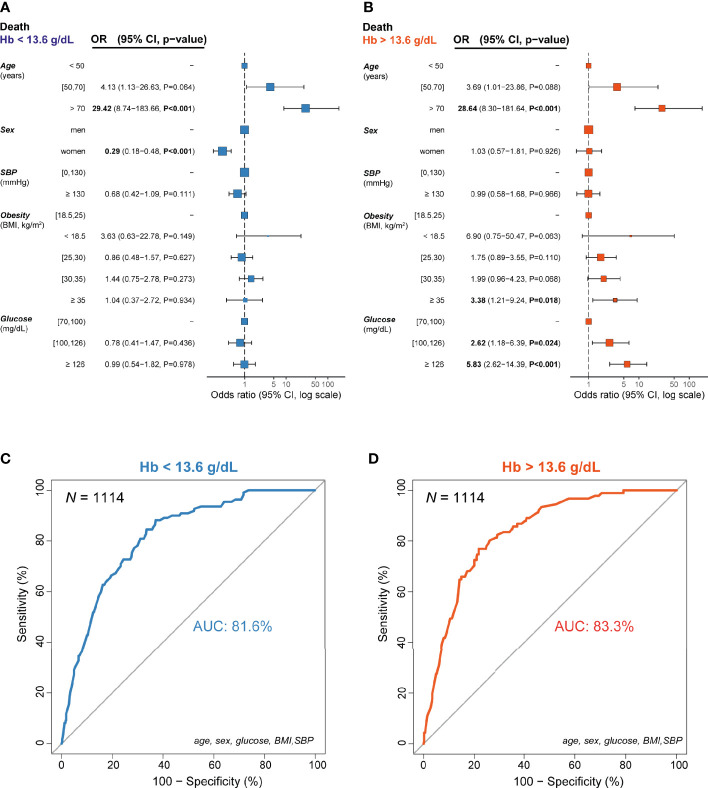
Incidence odds ratio of mortality from COVID-19 for the final models (*n*=1,114) according to the median hemoglobin concentrations (13.6 g/dL). **(A)** In individuals with hemoglobin levels below the median, **(B)** In individuals with hemoglobin levels above the median, **(C)** Receiver operating characteristic curve for the logistic regression model in individuals with hemoglobin levels below the median, **(D)** Receiver operating characteristic curve for the logistic regression model in individuals with hemoglobin levels above the median. Data are presented as odds ratio (OR) and 95% confidence intervals (CI). The reference groups (OR=1) for each variable are shown as “-”.: The odds ratios shown are adjusted through a logistic regression model which includes all variables listed. Data are also represented as a forest plot. AUC, area under the curve.

Considering that, apart from age, glucose was the strongest mortality predictor in our logistic regression models and the strong impact of hemoglobin on glucose modulating effects on prognosis, we built two final predictive models using the subset of patients with information about all clinical variables (*n*=1,114, one for hemoglobin concentrations below- and another one for above the median; cut-off =13.6 g/dL) (**Figure 5**). In the model for hemoglobin values above the median, glucose was a significant predictor of COVID-19 mortality (**Figure 5A**), whereas in the hemoglobin model for concentrations below the median, glucose concentrations had no prognostic value (**Figure 5B**). Notably, in subjects with hemoglobin levels above the median, women had the same likelihood of COVID-19 mortality than men. Thus, female sex was not a protective factor in individuals with hemoglobin concentrations above the median. Again, morbidly obese patients had a higher mortality risk than non-obese subjects only when hemoglobin concentrations were above the median. These mortality prediction models had an AUCROC of 81.6% and 83.3%, respectively (**Figures 5C, D**). We also checked for potential interactions between some predictors in these models and found no significant interactions. For the model with hemoglobin levels below the median, we did not find any interaction between age and sex (P=0.262), age and SBP (P=0.286), age and BMI (P=0.880), age and glucose (P=0.740), glucose and SBP (P=0.413), glucose and BMI (P=0.722), or BMI and SBP (P=0.942). Similarly, no interactions were found for the model with hemoglobin levels above the median for age and sex (P=0.155), age and SBP (P=0.268), age and BMI (P=0.822), age and glucose (P=0.433, glucose and SBP (P=0.825), glucose and BMI (P=0.876), or BMI and SBP (P=0.076).

We finally performed an external validation of these models using a test set of *n*=331 not used in training the final models. These patients had available data on hemoglobin levels but not on other parameters included in the machine learning analyses such as bilirubin, hs-CRP, sodium or platelets. In the case of the model for low hemoglobin, we obtained a classification prediction accuracy of 78%, whereas the prediction accuracy for the high hemoglobin model was 81%. The corresponding confusion matrices are shown in **Tables 2** and **3**. We also checked whether the results of these models could be affected by potential confounding factors known to alter the hemoglobin levels (smoking, COPD or dehydration based on hematocrit) or influencing liver steatosis (measured by alanine aminotransferase, ALT). The inclusion of these variables in the final models did not change the results (**Supplementary Figures 3** and **4**). Thus, in subjects with hemoglobin levels above the median, hyperglycemia was a strong independent predictor of mortality associated with COVID-19, while female sex lost its protective effect. Conversely, in subjects with hemoglobin levels below the median, women were less likely to die because of COVID-19 but hyperglycemia had no prognostic value.

**Table 2:** Confusion matrix for the model with hemoglobin levels below the median (<13.6 mg/dL).

		Actual class		Total	
		Death	Survival		
**Predicted** **class**	**Death**	TP=122	FP=30	152	PPV=80%
	**Survival**	FN=7	TN=9	16	NPV=56%
	**Total**	129	39	168	
		TPR=94%	TNR=23%		**Accuracy** 78%

TP, true positive; FP, false positive; FN, false negative; TN, true negative; PPV, Positive Predictive Value Precision; TPR, True Positive Rate Sensitivity; TNR, True Negative Rate, Specificity; NPV, Negative Predictive Value.

**Table 3:** Confusion matrix for the model with hemoglobin levels above the median (>13.6 mg/dL).

		Actual class		Total	
		Death	Survival		
**Predicted** **class**	**Death**	TP=120	FP=16	136	PPV=88%
	**Survival**	FN=15	TN=11	26	NPV=42%
	**Total**	135	27	162	
		TPR=89%	TNR=41%		**Accuracy** 81%

TP, true positive; FP, false positive; FN, false negative; TN, true negative; PPV, Positive Predictive Value Precision; TPR, True Positive Rate Sensitivity; TNR, True Negative Rate, Specificity; NPV, Negative Predictive Value.

In order to present the final models in a useful and user-friendly format for the clinical practice, we implemented the models as an interactive web application using html/CSS and Java Script at http://isvgirona.net/sarscov2/20201110/. The clinician only has to introduce the patient’s age, sex, SBP, BMI, glucose, and hemoglobin values and it internally categorizes the variables and uses the model corresponding to hemoglobin concentrations below or above the median to predict the probability of mortality.

## 4 Discussion

The results from this multi-center study of five different hospitals and Primary Health Care centers from Italy and Spainconfirm previous observations that both elevated blood glucose levels and higher BMI are associated with worse COVID-19 related outcomes. However, most of these studies have not taken into account the interaction of both parameters at the same time. Thus, several studies assessing obesity effects did not include comorbidities, such as diabetes, while studies that found associations with hyperglycemia did not consider obesity as a confounding factor (12–16). A recent review about prediction models for the diagnosis and prognosis of COVID-19 identified 23 models for the estimation of mortality risk (25). Several of these models included comorbidities. However, only one of these 23 models considered the joint role of obesity and diabetes in modifying COVID-19 outcomes (26). Thus, Bello-Chavolla et al. found that, in a Mexican population, early-onset diabetes increased the risk of COVID-19-related mortality, while obesity mediated 50% of the effects of diabetes on COVID-19 lethality and conferred an increased risk for ICU admission. Here, we have found that, when both obesity and hyperglycemia are considered together, although subjects with obesity and hyperglycemia had an increased likelihood of admission to the ICU, only the latter was an independent predictor of mortality.Notably, these results were replicated indepenently in two different countries. We also observed that both extremes of BMI were associated with a poorer prognosis. This is in line with a recent population study in England in which BMI had a U shape relation to mortality in patients with type 1 and type 2 diabetes (27).

Modern machine learning methods, such as random forest, are promising computational approaches for feature selection in predictive modeling that are increasingly applied to clinical problems. Machine Learning tree-based algorithms are particularly well-suited to this aim. In contrast to classical linear model-based statistical methods, they are fully non-parametric model-free methods that can capture complex dependency patterns within the datasets affecting the phenotype. They provide variable importance measures that can be used to identify relevant features. In addition, they are invariant to monotonic transformations, have a good performance in non-linear datasets and can auto-correct dependencies among variables, thereby rendering them as particularly suitable to deal with complex datasets. Notably, tree-based variable selection methods have shown to perform better than classic regression-based methods in large, multicenter datasets (28). Therefore, we also performed machine learning analyses including clinical parameters such as plasma CRP, creatinine, platelets levels, and iron-related parameters (hemoglobin and bilirubin).

The most frequently used variables in mortality risk models included comorbidities, age, sex, lymphocyte counts, CRP and creatinine (25). Renal impairment (9, 16, 27, 29), plasma CRP concentration (9, 27, 29) and blood platelet count have been previously reported to be associated with a poorer prognosis in patients with COVID-19 and we here confirm their independent associations in a Machine Learning model. Both hemoglobin and bilirubin were also associated with COVID-19 mortality. In a recent meta-analysis that identified more than 30 risk factors associated with a higher risk of severe COVID-19, lower levels of hemoglobin but increased serum ferritin, among others, were associated with severe COVID-19 (29). These results were confirmed in an independent meta-analysis (30). Compared to moderate cases, severe COVID-19 cases had lower hemoglobin [weighted mean difference (WMD), - 4.08 g/L (95% CI - 5.12; - 3.05)] and higher ferritin [WMD, - 473.25 ng/mL (95% CI 382.52; 563.98)]. A significant difference in mean ferritin levels of 606.37 ng/mL (95% CI 461.86; 750.88) was found between survivors and non-survivors, but not in hemoglobin levels (30). Similarly, low levels of hemoglobin have been recently shown to be predictive factors for the diagnosis and prediction of patients with COVID-19 (17, 18). Hemoglobin levels have also shown to predict the severity of COVID-19, with a gradual decrease with disease progression (19). In addition, anemia has been associated with severe COVID-19 (21)and has also been identified as a single independent predictor of mortality in COVID-19 patients (20, 22). Finally, in a recent meta-analysis of 83 studies, total bilirubin (mean difference 2.08 mmol/L, 95% CI 1.36-2.80 mmol/L; P<0.001) was also observed to be increased in patients with severe compared to non-severe COVID-19 patients (31). In agreement with these previous results, we also found that hemoglobin levels below the median were associated with a higher risk of mortality due to COVID-19 in men. However, we did not find significant differences in women.

Intriguingly, we found that the glucose predictive value for COVID-19 mortality was strongly modulated by hemoglobin levels. Despite anemia is considered to be an independent predictor of mortality due to COVID-19 (20)and hemoglobin was negatively associated with COVID-19 mortality in men in our study, we found that high hemoglobin levels had a strong impact on modulating the prognostic value of hyperglycemia in relation to COVID-19 mortality. Hence, men with moderate and severe hyperglycemia had a strikingly increased mortality risk due to COVID-19 compared to normoglycemic individuals only when they also had increased hemoglobin levels. Women with moderate and severe hyperglycemia and increased hemoglobin concentrations also showed a trend towards a higher mortality risk compared to women with normal glucose concentrations. As the number of women with hemoglobin levels above the median was substantially smaller than that of men, it is likely that increasing the number of women would also result in significant associations. In any case, when both men and women were included, we also found that glucose was a significant predictor of COVID-19 mortality in subjects with increased hemoglobin levels. Remarkably, gender had no effect on mortality in these subjects. Thus, women were not more protected from COVID-19 mortality than men when hemoglobin concentrations were above the median. Our results, are in line with a recent study including 9,467 hospitalized COVID-19 patients showing that patients with hemoglobin ≥ 16 g/dL had significantly higher adjusted in-hospital mortality compared to patients with hemoglobin between 12 and 14 g/dL [OR (95% CI): 1.62 (1.15-2.27), *P*=0.005]. As SARS-CoV-2 infection increases coagulopathy (32), high hemoglobin levels may be associated with and hypercoagulable state leading to systemic thrombosis.

A viral interaction with hemoglobin molecule, through ACE2, CD147, CD26 and other receptors located on erythrocytes and/or blood cell precursors, has been highlighted (33, 34). Certain viral proteins could attack the 1-beta chain of hemoglobin to dissociate iron, generating dysfunctional hemoglobin with reduced oxygen transport capacity (35). SARSCoV-2 has been suggested to invade host cells *via* CD147-spike protein interaction (36). As there are about 3000 molecules of CD147 per erythrocyte, the entry of SARS-CoV-2 through this pathway has been considered a possible basic pathogenic mechanism. In fact, SARS-CoV was previously shown to interfere with hemoglobin at erythrocyte and bone marrow level (34). In a recent study, Lancman *et al.* found that from 38 hospitalized COVID-19 patients, 80% had elevated plasma-free hemoglobin levels (> 5 mg/dL), a marker of hemolysis. Viral spike protein interaction with CD147 on red blood cells could be a potential mechanism of this intravascular hemolysis.

Regarding the possible pathophysiology mediating all these effects, hyperglycemia itself may up-regulate the expression of ACE2, facilitating the entry of the virus into cells (37) leading to a poorer prognosis. It could be possible that the co-existence of hyperglycemia and increased hemoglobin levels amplify the entry of SARS-CoV-2 in blood cells, facilitating its intracellular replication. This could partly explain the strong impact of glucose on COVID-19 mortality that we observed in men with hemoglobin concentrations above the median. Importantly, none of the 23 models published so far to predict COVID-19 mortality has included hemoglobin levels.

### 4.1 Strengths and Limitations

This study has several strengths. The findings in this multicenter and diverse study in five different hospitals and the Primary Health Care system have been homogenous. This allowed the replication of the importance of hyperglycemia across the different centers. This study also has potential limitations. There is missingness for some variables, but this is randomly distributed across the centers. Further, we may have missed deaths that occurred outside the hospital. There is also a relative over-representation of in-hospital patients (36% of all subjects) while the number of subjects requiring hospitalization is far lower when considering asymptomatic patients, so the finding may be only applicable to the most severe patients. However, even taking into account this limitation, the targeting of glycemia and hemoglobin concentrations in these severe patients seems of utmost importance.

In summary, hemoglobin levels had a strong impact on hyperglycemia and morbid obesity prognostic value. Only morbidly obese subjects or subjects with hyperglycemia and hemoglobin concentrations above the median were at increased mortality risk. Considering the strong impact of glucose and morbid obesity on COVID-19 mortality, monitoring hemoglobin levels in subjects with hyperglycemia is thus of utmost importance. Low hemoglobin levels have already been identified as an independent predictor of mortality due to COVID-19. In the present study, we add additional valuable information regarding the role of hemoglobin in COVID-19 and extend these findings to high hemoglobin levels. We provide an online calculator that may have utility in the clinical settings to help clinicians identify and prioritize those patients at higher risk of death. To our knowledge, this is the first study to analyze different independent samples from different institutions from two countries and to use machine learning tools for the analysis.

## Obesity and Type 2 Diabetes COVID-19 Study Group

Mariona Reixach Fumaña (Service of Internal Medicine Dr. Josep Trueta University Hospital, Catalonia, Spain), Susana Jimenez-Murcia (Department of Psychiatry, Hospital Universitari de Bellvitge-IDIBELL and CIBERobn, Hospitalet de Llobregat, Barcelona, Spain), Mikel Eatxandi (Department of Psychiatry, Hospital Universitari de Bellvitge, Hospitalet de Llobregat, Barcelona, Spain); Alexander Rombauts and Gabriela Abelenda-Alonso (Department of Infectious Diseases, Hospital Universitari de Bellvitge, Bellvitge Biomedical Research Institute (IDIBELL), Barcelona, Spain); Caterina Guidone (Hospital of Bologna, Italy); Danila Anello (Hospital of Bologna, Italy); Giulia Giannetti (Hospital of Bologna, Italy).Emilio Ortega (Department of Endocrinology & Nutrition, Diabetes Unit. Hospital Clínic Barcelona, Spain, Biomedical Research NetworkingCenter for Physiopathology of Obesity and Nutrition (CIBEROBN), Madrid, Spain Institut d’investigacions biomèdiques August Pi i Sunyer (IDIBAPS), Barcelona, Spain) Ignacio Conget (Department of Endocrinology &Nutrition, Diabetes Unit. Hospital Clínic Barcelona, Spain, Biomedical Research NetworkingCenter for Physiopathology of Obesity and Nutrition (CIBEROBN), Madrid, Spain Institut d’investigacions biomèdiques August Pi i Sunyer (IDIBAPS), Barcelona, Spain); Clara Viñals (Department of Endocrinology &Nutrition, Diabetes Unit. Hospital Clínic Barcelona, Spain); Carmen Hernández-Aguado (Department of Anesthesia; Dr. Josep Trueta University Hospital, Catalonia, Spain); Josep-Maria Sirvent (Intensive Care Department, Dr. Josep Trueta University Hospital, Catalonia, Spain), Ramon Orriols (Department of Pneumology; Dr. Josep Trueta University Hospital, Catalonia, Spain);Mercé Fernández-Balsells (Department of Endocrinology, Dr. Josep Trueta University Hospital, Catalonia, Spain); Wifredo Ricart (Dr. Josep Trueta University Hospital, Catalonia, Spain).

## Data Availability Statement

The raw data supporting the conclusions of this article will be made available by the authors, without undue reservation.

## Ethics Statement

The protocol was approved by the Ethical Committee of the Fondazione Policlinico Universitario A. Gemelli IRCCS, Catholic University, Rome, Italy with Approval Number: 0014355/20. Before enrolment, each subject gave informed consent. ClinicalTrials.gov ID: NCT04324684. The protocol was also independently approved by the ethics committee of the Hospital of Bologna, Hospital of Bellvitge, Hospital Clínic, Hospital of Girona and the Primary Care Ethics Committee. The patients/participants provided their written informed consent to participate in this study.

## Author Contributions

JM-P analysed the data. JMP-P and JF-R wrote the manuscript. All authors provided data and contributed to the discussion. JF-R takes the responsibility for the contents of the article. All authors contributed to the article and approved the submitted version.

## Funding

We thank CERCA Programme/Generalitat de Catalunya for institutional support. This study was in part supported by the Spanish Plan Nacional de I+D+i 2013‐2016 and the Instituto de Salud Carlos III, Subdirección General de Redes y Centros de Investigación Cooperativa, Ministerio de Economía, Industria y Competitividad, Spanish Network for Research in Infectious Diseases (REIPI RD16/0016/0001) ‐ co-financed by European Development Regional Fund “A way to achieve Europe”, Operative Programme Intelligent Growth 2014‐2020. CIBERobn is an initiative of ISCIII. Partially supported by FIS Grant (PI17/01167), ISCIII. JM-P is funded by the Miguel Servet Program from the Instituto de Salud Carlos III (ISCIII CP18/00009), co-funded by the European Social Fund “Investing in your future”.

## Conflict of Interest

The authors declare that the research was conducted in the absence of any commercial or financial relationships that could be construed as a potential conflict of interest.

## Publisher’s Note

All claims expressed in this article are solely those of the authors and do not necessarily represent those of their affiliated organizations, or those of the publisher, the editors and the reviewers. Any product that may be evaluated in this article, or claim that may be made by its manufacturer, is not guaranteed or endorsed by the publisher.

## Supplementary Material

Supplementary Figure 1 Incidence odds ratio of mortality and ICU admission from COVID-19 by potential prognostic variables in hospital patients by country. **(A)** Mortality in Spain, **(B)** ICU admission in Spain, **(C)** Mortality in Italy, **(D)** ICU admission in Italy. Data are presented as odds ratio (OR) and 95% confidence intervals (CI). The reference groups (OR=1) for each variable are shown as “-”.The odds ratios shown are adjusted through a logistic regression model which includes all variables listed. Data are also represented as a forest plot.

Supplementary Figure 2 Incidence odds ratio of mortality from COVID-19 by potential prognostic variables in men according to iron-related parameters. **(A)** men with hemoglobin concentrations below the median, **(B)** men with hemoglobin concentrations above the median, **(C)** men with bilirubin concentrations below the median, **(D)** men with bilirubin concentrations above the median. Data are presented as odds ratio (OR) and 95% confidence intervals (CI). The reference groups (OR=1) for each variable are shown as “-”.: The odds ratios shown are adjusted through a logistic regression model which includes all variables listed. Data are also represented as a forest plot. Iron parameters were dichotomized based on the median.

Supplementary Figure 3 Incidence odds ratio of mortality from COVID-19 including smoker status and alanine aminotransferase (ALT) as potential confounder variables in the final models according to the median hemoglobin concentration (13.6 g/dL). **(A, B)** Models including smoker status (*n*=878) as a confounding factor in subjects with hemoglobin levels below and above the median, respectively. **(C, D)** Models including liver function assessed by alanine aminotransferase (ALT, *n*=1333) as a confounding factor in subjects with hemoglobin levels below and above the median, respectively.

Supplementary Figure 4 Incidence odds ratio of mortality from COVID-19 including chronic obstructive pulmonary disease (COPD) and hematocrit as potential confounder variables in the final models according to the median hemoglobin concentration (13.6 g/dL). **(A, B)** Models including chronic obstructive pulmonary disease (COPD, *n*=422) as a confounding factor in subjects with hemoglobin levels below and above the median, respectively. **(C, D)** Models including hematocrit (*n*=926) as a confounding factor in subjects with hemoglobin levels below and above the median, respectively.
