# Supplementary figures and images for: Blood Hemoglobin Substantially Modulates the Impact of Gender, Morbid Obesity, and Hyperglycemia on COVID-19 Death Risk: A Multicenter Study in Italy and Spain
Source: Front Endocrinol (Lausanne). 2021 Nov 2;12:741248. doi: 10.3389/fendo.2021.741248 (PMC8593102; doi:10.3389/fendo.2021.741248)

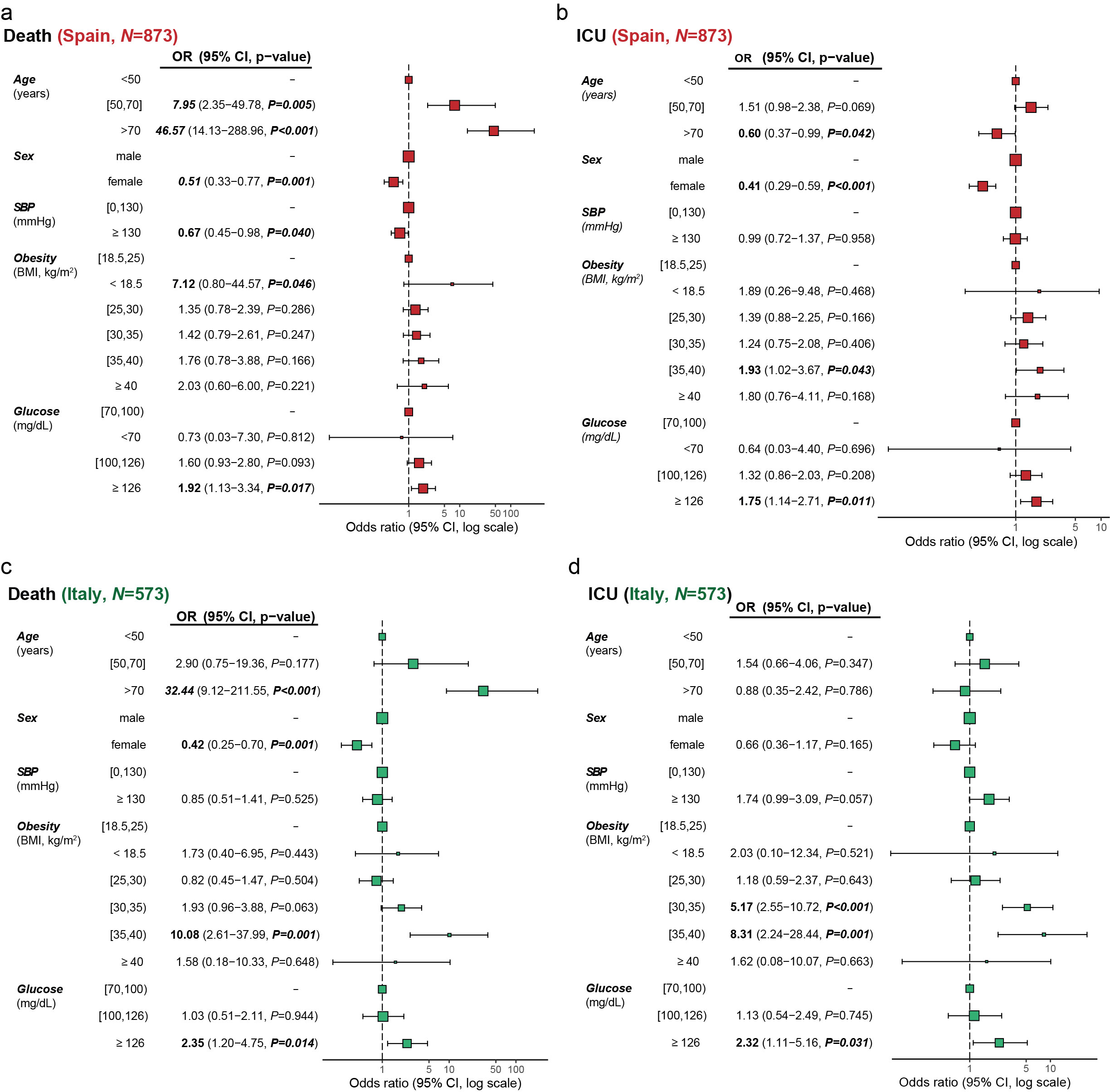

Supplement: Supplementary Figure 1 — Incidence odds ratio of mortality and ICU admission from COVID-19 by potential prognostic variables in hospital patients by country. (A) Mortality in Spain, (B) ICU admission in Spain, (C) Mortality in Italy, (D) ICU admission in Italy. Data are presented as odds ratio (OR) and 95% confidence intervals (CI). The reference groups (OR=1) for each variable are shown as “-”.The odds ratios shown are adjusted through a logistic regression model which includes all variables listed. Data are also represented as a forest plot. [file Image_1.jpg]

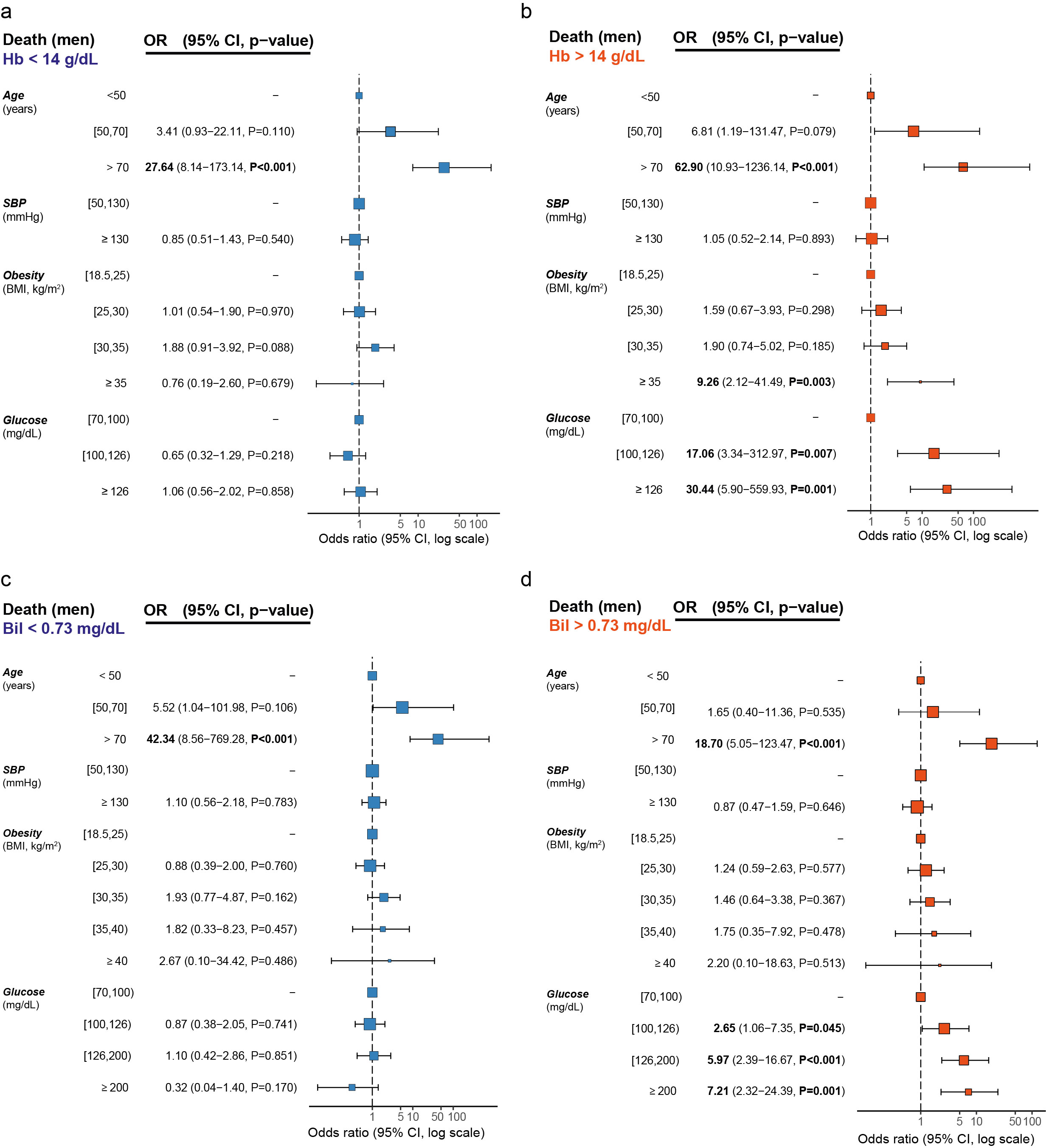

Supplement: Supplementary Figure 2 — Incidence odds ratio of mortality from COVID-19 by potential prognostic variables in men according to iron-related parameters. (A) men with hemoglobin concentrations below the median, (B) men with hemoglobin concentrations above the median, (C) men with bilirubin concentrations below the median, (D) men with bilirubin concentrations above the median. Data are presented as odds ratio (OR) and 95% confidence intervals (CI). The reference groups (OR=1) for each variable are shown as “-”.: The odds ratios shown are adjusted through a logistic regression model which includes all variables listed. Data are also represented as a forest plot. Iron parameters were dichotomized based on the median. [file Image_2.jpg]

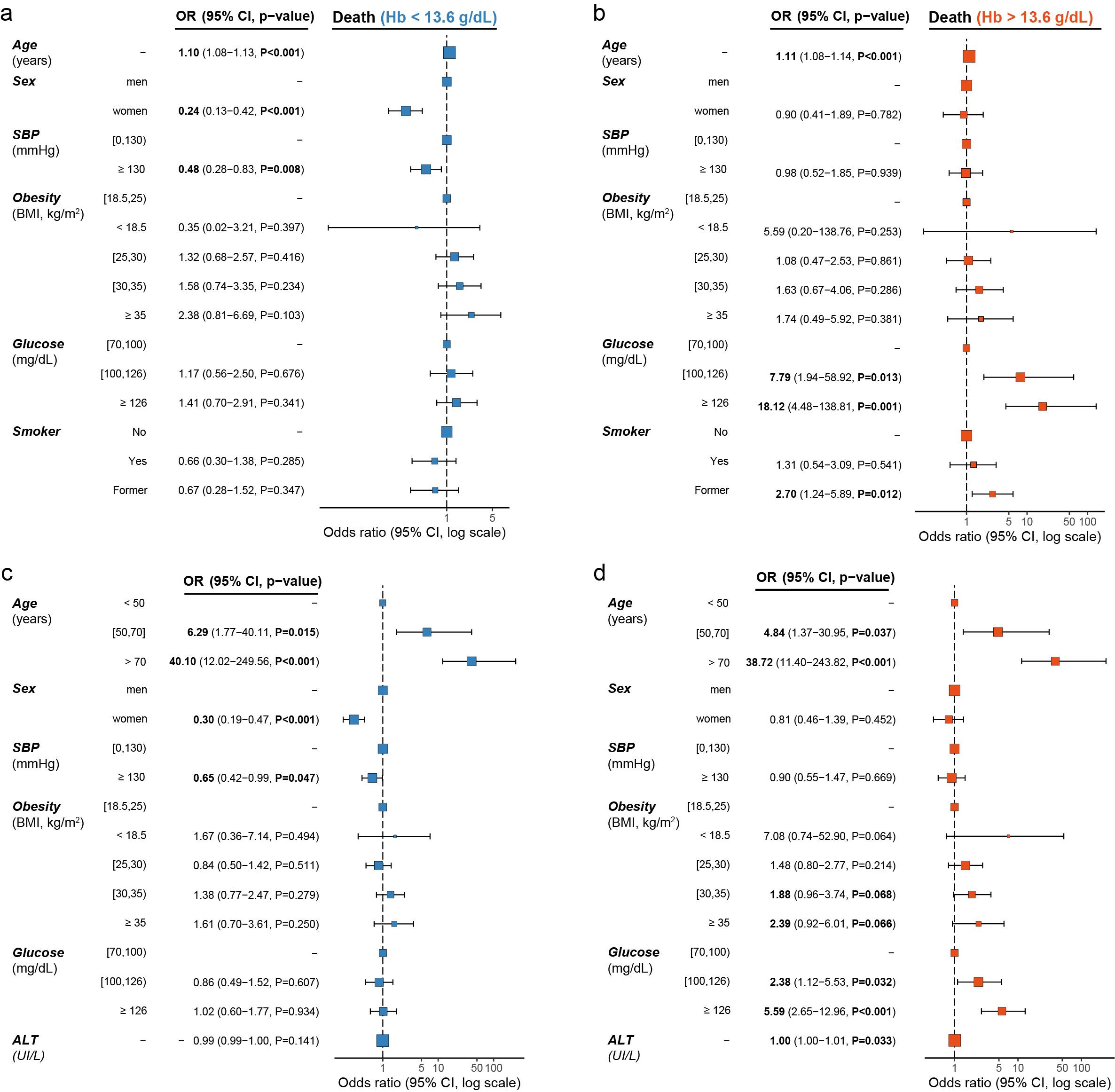

Supplement: Supplementary Figure 3 — Incidence odds ratio of mortality from COVID-19 including smoker status and alanine aminotransferase (ALT) as potential confounder variables in the final models according to the median hemoglobin concentration (13.6 g/dL). (A, B) Models including smoker status (n=878) as a confounding factor in subjects with hemoglobin levels below and above the median, respectively. (C, D) Models including liver function assessed by alanine aminotransferase (ALT, n=1333) as a confounding factor in subjects with hemoglobin levels below and above the median, respectively. [file Image_3.jpg]

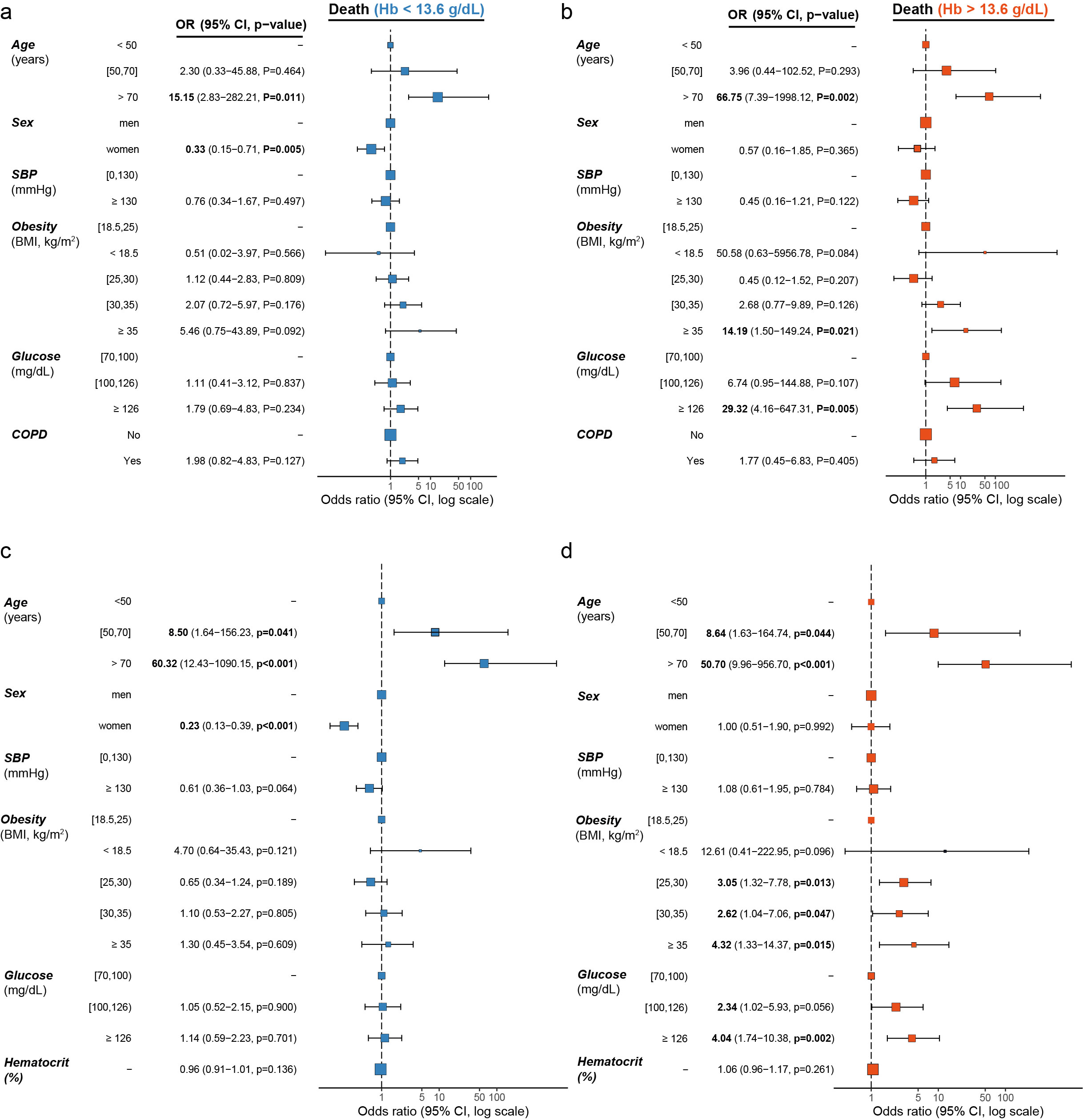

Supplement: Supplementary Figure 4 — Incidence odds ratio of mortality from COVID-19 including chronic obstructive pulmonary disease (COPD) and hematocrit as potential confounder variables in the final models according to the median hemoglobin concentration (13.6 g/dL). (A, B) Models including chronic obstructive pulmonary disease (COPD, n=422) as a confounding factor in subjects with hemoglobin levels below and above the median, respectively. (C, D) Models including hematocrit (n=926) as a confounding factor in subjects with hemoglobin levels below and above the median, respectively. [file Image_4.jpg]
